# Micro-Injection Molding and Debinding Behavior of Hydroxyapatite/Zirconia Bi-Materials Fabricated by Two-Component Micro-Powder Injection Molding Process

**DOI:** 10.3390/ma16196375

**Published:** 2023-09-24

**Authors:** Al Basir, Norhamidi Muhamad, Abu Bakar Sulong, Muhammad bin Mohamed Amin, Nashrah Hani Jamadon, Nabilah Afiqah Mohd Radzuan

**Affiliations:** Department of Mechanical and Manufacturing Engineering, Faculty of Engineering and Built Environment, Universiti Kebangsaan Malaysia, Bangi 43600, Selangor, Malaysia; al.basir005@yahoo.com (A.B.); norhamidi@ukm.edu.my (N.M.); muhammadqx@gmail.com (M.b.M.A.); nashrahhani@ukm.edu.my (N.H.J.); afiqah@ukm.edu.my (N.A.M.R.)

**Keywords:** two-component micro-powder injection molding, HA/3YSZ micro-parts, feedstock, solvent debinding, thermal debinding

## Abstract

The micro-scale joining of two different materials using two-component micro-powder injection molding (2C-µPIM) is an intriguing technique. The formation of defects in bi-materials at different processing stages makes this technique challenging. This study presents the fabrication of defect-free bi-material micro-parts containing hydroxyapatite (HA) and 3 mol% yttria-stabilized zirconia (3YSZ) via 2C-µPIM. Critical powder volume concentrations (CPVCs) of 61.7 vol% and 47.1 vol% were obtained for the HA and 3YSZ powders, respectively. Based on the CPVCs, the optimal loadings for the HA and 3YSZ powders were selected as 60 vol% and 45 vol%, respectively. The HA and 3YSZ feedstocks were prepared by separately mixing the optimal powder contents with low-density polyethylene (LDPE) and palm stearin binders. The feedstocks displayed pseudoplastic behavior, and the lowest ranges of viscosity for the HA and 3YSZ at a temperature of 180 °C were 157.1–1392.5 Pa·s and 726.2–985.5 Pa·s, respectively. The feedstocks were injected to produce green HA/3YSZ micro-sized components. It was found that a solvent debinding temperature of 70 °C removed 60.6% of the palm stearin binder from the sample. In the thermal debinding stage, the open channels that formed in the bi-material sample’s solvent debound at 70 °C and contributed to the removal of 93 to 95% of the binder system. When the debound bi-materials were sintered at 1300 °C, the highest relative density of 96.3% was obtained. The sintering operation revealed a linear shrinkage between 13 and 17% in the sintered HA/3YSZ micro-parts.

## 1. Introduction

The significance of the mass production of intricately downsized or micro-structured components has intensified in recent years. Micro-powder injection molding (µPIM), which is a modification of the powder injection molding (PIM) process, is a financially viable method of processing materials for the fabrication of ceramic and metallic micro-sized components [1,2,3]. Similarly to PIM, the µPIM process offers several advantages such as low production cost, excellent surface finishes, nominal post-production scrap, and outstanding mechanical properties [4,5,6,7,8,9]. In recent times, the perceptions of the global market toward micro-sized components have transformed immensely, and the modern view is to incorporate a variety of operational competencies into a single micro-component. Accordingly, µPIM was altered to a two-component micro-powder injection molding (2C-µPIM) process, which allows two dissimilar materials to be joined on a micro-scale to yield bi-material micro-components that possess multiple functionalities [10,11,12]. The benefits of affordable production and versatility in the selection of materials have made 2C-µPIM a phenomenal manufacturing method in the current era.

**Figure 1:**
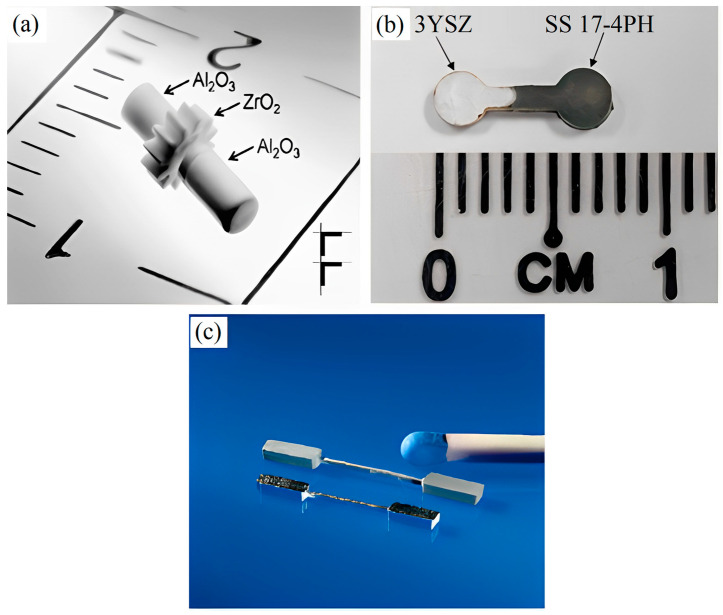
Micro-parts fabricated using 2C-µPIM process: (**a**) shaft-to-collar connection of ZrO_2_/Al_2_O_3_, (**b**) bi-materials of 3YSZ/SS 17-4PH, and (**c**) bi-metals of SS 316L/SS 17-4PH, reused with permission from John Wiley and Sons and Elsevier [13,14,15].

Ruh et al. [13] used the 2C-µPIM technique to fabricate a shaft-to-collar connection employing two different ceramic powders, namely alumina (Al_2_O_3_) and zirconia (ZrO_2_), with the intention of using it in micromechanics or medical devices (Figure 1a). Basir et al. [14] used 2C-µPIM to produce micro-sized bi-material components containing 3 mol% of yttria-stabilized zirconia (3YSZ) and stainless steel 17-4PH (SS 17-4PH) to examine their viable use in multiple micro-applications (Figure 1b). Imgrund et al. [15] reported the fabrication of bi-metals of stainless steel 316L (SS 316L) and SS 17-4PH by means of the 2C-µPIM process (Figure 1c). Basically, the investigation regarding 2C-µPIM is currently in its inception stage, and it will have to prove its ability to fabricate flawless components if it is to compete in the extremely ambitious worldwide market.

The four main steps of 2C-µPIM are mixing, micro-injection molding, debinding, and sintering. The process typically begins with the mixing step, whereby homogeneous feedstocks are fabricated by mixing two distinct kinds of powder particles discretely with a multi-component binder system. Each feedstock type is then injected, either sequentially or simultaneously, into the molding apparatus to yield green micro-sized bi-material parts. After the micro-injection molding procedure, the employed binder system is eliminated from the micro-samples using solvent and thermal debinding methods. The debound micro-sized samples are finally sintered to produce the required mechanical and physical characteristics [11].

A variety of restraints can be introduced through the use of fine and larger-sized powder particles [16]. A major difficulty is thought to be an increase in the viscosity of the feedstock in light of a reduction in the size of the powder particles [17]. A high packing density may be difficult to achieve with fine powders due to particle agglomeration. It is also possible that the debinding process will take a longer time if the capillary pathways within the particles are reduced in size [18,19]. On the other hand, the likelihood of deformation increases with the use of larger-sized powder particles because of a reduction in the debinding strength. Since larger particles have less particle-to-particle interaction per unit volume than smaller particles, when they are packed together, their inherent strength is lower than that of smaller particles [20]. So far, only a few PIM researchers have used powder particles that are larger than 30 µm [21]. During the 2C-µPIM process, the fluidity of two different feedstocks to fill the tiny cavity of the mold entirely is ascertained by the binder system. Until the initiation of the sintering process, the shapes of the micro-sized bi-materials are also maintained by the binders. The key characteristics of effective binders include non-toxicity, cost-effectiveness, outstanding flow behavior, excellent powder-binder interaction, and favorable binder extraction features [22,23]. In the mixing step of the 2C-µPIM process, it is essential to prepare a balanced powder–binder mixture. This is because an elevated content of powder increases the feedstock’s viscosity but makes mixing challenging, while a decreased content of powder increases shrinkage in the sintered samples [24,25]. It has been reported that preparing feedstock based on the optimal powder loading results in sintered micro-parts with negligible defects and better mechanical properties [26]. During injection molding, the injection parameters must be carefully selected so as to obtain samples without any defects. Another crucial aspect of the injection process is the demolding of the green bi-material micro-components, since such delicate components can be distorted during the ejection process. After the micro-injection molding process, the primary purpose of debinding is to rid the bi-material micro-parts of the binders without detrimentally affecting bonding. When the solvent in metal/ceramic-based micro-parts that are bi-materials is debound, bonding failure occurs due to the use of a very high solvent debinding temperature [14]. Therefore, it is important to select the debinding temperature and time carefully during the solvent and thermal debinding procedures to achieve defect-free debound micro-samples. During sintering, appropriate sintering parameters such as the sintering temperature, heating rate, holding time, and cooling rate need to be selected to achieve sintered micro-sized bi-materials that are free of defects in the bonding region.

In the current study, hydroxyapatite (HA) and 3 mol% yttria-stabilized zirconia (3YSZ) ceramics were selected as the materials. HA is well-known for its outstanding biocompatibility, excellent compressive strength, and corrosion resistance. Moreover, it contains attributes that support the osseointegration and formation of new bone [27,28]. On the other hand, 3YSZ has attracted the attention of the scientific community due to its features of good biocompatibility, excellent wear resistance, good flexural strength, chemical stability, and high temperature stability [29,30]. The joining of HA and 3YSZ ceramics at the micro level is considered to be a great choice for biomedical applications. The binder system of this present study combined low-density polyethylene (LDPE) and palm stearin, as they are a popular choice for PIM due to their excellent technical characteristics, cost-effectiveness, and availability.

This present study aimed to successfully use the 2C-µPIM process to produce green bi-material micro-sized components of HA and 3YSZ using HA and 3YSZ ceramic powders. It also examined methods of removing the binder system from components that are green to produce parts post-sintering.

## 2. Materials and Methods

**Figure 2:**
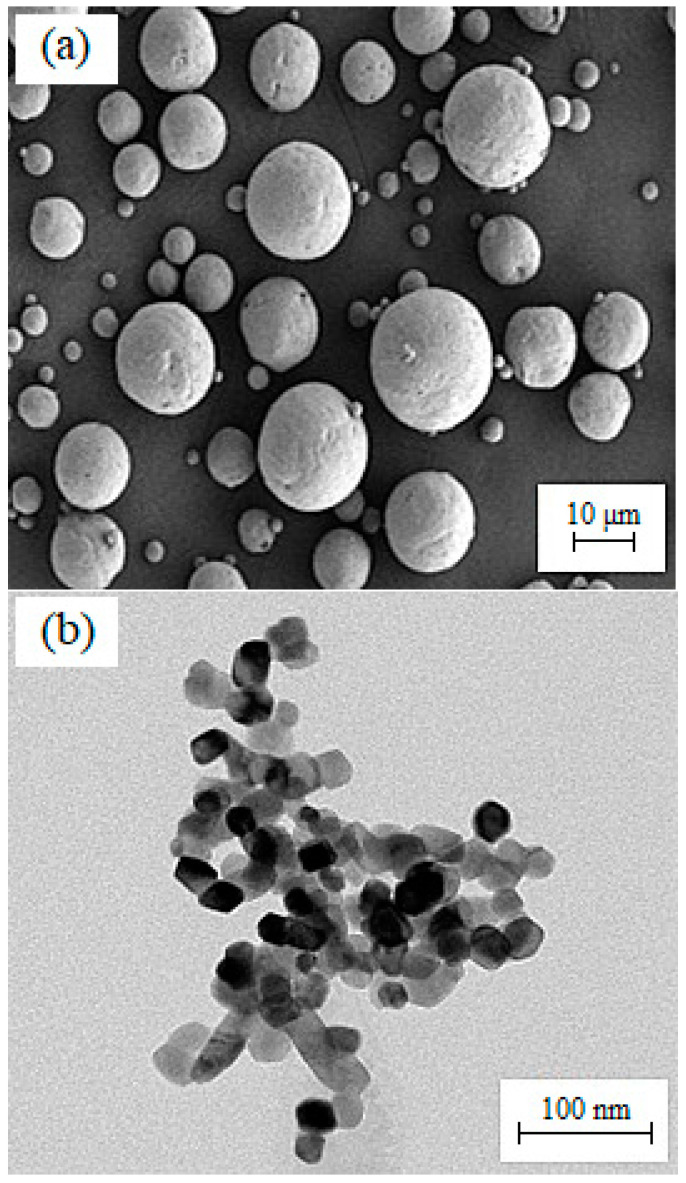
Morphology of the powders: (**a**) FESEM image of HA particles, and (**b**) TEM image of 3YSZ particles.

This present study used HA and 3YSZ ceramic powders with mean particle sizes of 1.7 μm and 172.4 nm, respectively. These HA and 3YSZ powders were purchased from Vistec Technology Services (Puchong, Malaysia) and Inframat Advanced Materials LLC (Manchester, CT, USA), respectively. The densities of the HA and 3YSZ powders, as determined by a pycnometer, were found to be 2.4831 and 5.6387 g/cm^3^, respectively, using an AccuPyc II 1340 gas displacement pycnometer. A ZEISS MERLIN Compact field emission scanning electron microscope (FESEM) was used to observe the morphology of the HA, while a Thermo Scientific Talos L120C transmission electron microscope (TEM) was used to examine that of the 3YSZ powder (Figure 2). The binder system consisted of 40 and 60 wt% of LDPE and palm stearin, respectively. The LDPE, which was the polymer backbone of the green sample, had a density of 0.91 g/cm^3^ and was obtained from Polyolefin Corporation (Singapore) Pte Ltd., while the palm stearin, which was the significant constituent, had a density of 0.891 g/cm^3^ and was obtained from Sime Darby Kempas Sdn. Bhd. As palm stearin has the capability to act as both a lubricant and surfactant, it was used to improve the flow properties [31], while LDPE was applied to enhance the strength of the green sample [32,33,34]. Arifin et al. [35] had previously used a binder system of polyethylene (PE) and palm stearin to fabricate HA/titanium alloy (Ti6Al4V) composites using PIM. This research work used a TA Instruments Q2000 differential scanning calorimeter (DSC) to determine the temperature at which the binders melted. Meanwhile, a PerkinElmer simultaneous thermal analyzer (STA) 6000 was used to determine the temperature at which the binders degraded. The mixing and debinding operations required a certain temperature, which was determined using the findings of the differential scanning calorimetry (DSC) and thermogravimetric analysis (TGA) as the standard [36,37,38]. Table 1 displays the different characteristics of the LDPE and palm stearin binders.

The oil absorption technique was used to determine the critical powder volume concentration (CPVC) of both the HA and 3YSZ. According to the critical powder loadings, the optimal powder loadings for HA and 3YSZ were chosen as 60 and 45 vol%, respectively. The feedstocks of the HA and 3YSZ were prepared by individually combining HA 60 vol% and 3YSZ 45 vol% with the binders in a Brabender W 50 EHT measuring mixer at 150 °C and a consistent blade rotational speed of 25 rpm. A mixing time of 2 h and 1 h in the mixer was employed to prepare the HA and 3YSZ feedstocks, respectively. A Shimadzu CFT-500D capillary rheometer was used to examine the behaviors of the HA and 3YSZ feedstocks at 160, 170, and 180 °C. These findings were important for anticipating the flow properties of the feedstocks as they filled the cavity of the mold.

**Figure 3:**
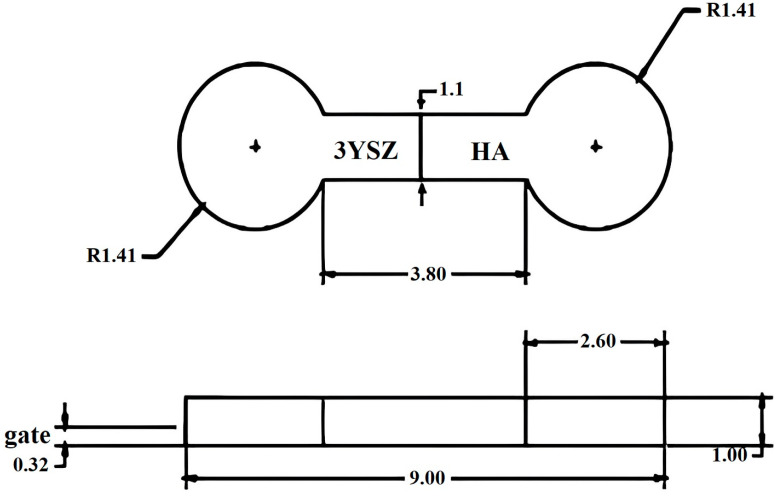
Dimensions of the HA/3YSZ micro-part (mm).

A semi-automatic Xplore Instruments injection molding 12 machine was used to produce the components that were green, bi-material, and micro-sized by processing the HA and 3YSZ feedstocks. The dimensions of the HA/3YSZ micro-component are demonstrated in Figure 3. The appropriate micro-injection molding conditions were applied to ensure that the samples were free of defects, including cracks, short shots, and flashing. Demolding was conducted meticulously immediately after the completion of the injection molding process and adequate cooling of the mold to avoid sample deformation.

**Figure 4:**
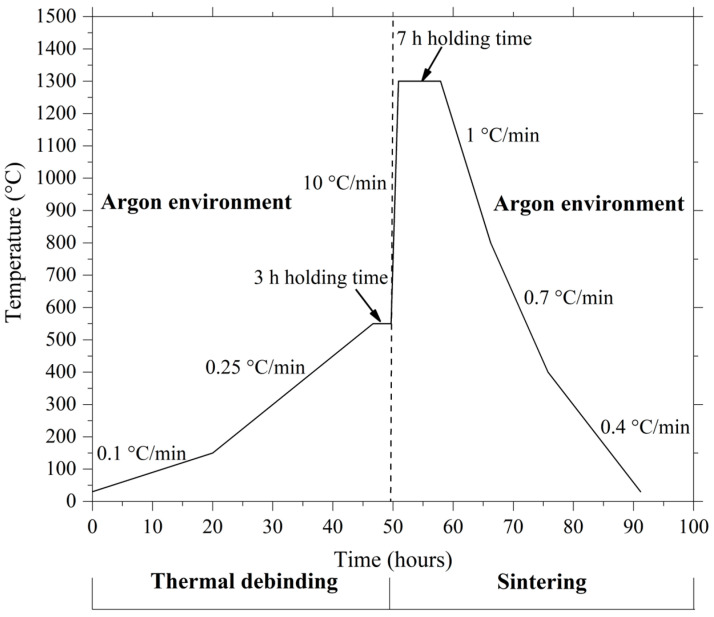
Consecutive thermal debinding and sintering profile.

An MMM Medcenter VentiCell 111 drying oven was utilized to perform the solvent debinding process. The green and micro-sized HA/3YSZ components were placed in an acetone submersion for 40 min at 50, 60, and 70 °C to eradicate the palm stearin binder. The solvent-debound HA/3YSZ micro-samples were then thermally debound to eradicate the LDPE and palm stearin remnants. The thermal debinding and sintering were performed successively in an HTF-15/200-60 tube furnace in an argon-rich atmosphere (Figure 4). In order to avoid the formation of defects in the micro-sized samples, an appropriate thermal debinding schedule was chosen based on a TGA evaluation. The solvent-debound HA/3YSZ micro-parts were heated, starting at room temperature and increasing at 0.1 °C/min until 150 °C. The temperature was eventually increased at 0.25 °C/min to 550 °C for 3 h to complete the thermal debinding operation. Five thermally debound samples that underwent the same conditions were analyzed to evaluate the removal of binders.

During sintering, the thermally debound HA/3YSZ micro-parts were heated at 10 °C/min, from 550 °C to 1300 °C, for 7 h (Figure 4). The sintering temperature was determined based on the findings of Basir et al. [14], who used 2C-µPIM to fabricate 3YSZ and SS 17-4PH bi-material components that were micro-sized, and Ramli et al. [39] who used PIM to fabricate SS 316L-HA composites. Three phases of cooling were applied to the HA/3YSZ micro-parts produced in the tube furnace. The sintered micro-sized bi-materials were first cooled to 800 °C at 1 °C/min before being further cooled from 800 to 400 °C at 0.7 °C/min. They were then cooled from 400 °C to room temperature at 0.4 °C/min. The density of the five HA/3YSZ micro-parts was determined employing the Archimedes method in accordance with MPIF standard 42 [40]. By comparing the length variations between micro-injection molded green parts and sintered parts in compliance with MPIF standard 44, the shrinkage percentage of the bi-materials was calculated [40].

## 3. Results and Discussion

### 3.1. Determining the Optimal Powder Loading

**Figure 5:**
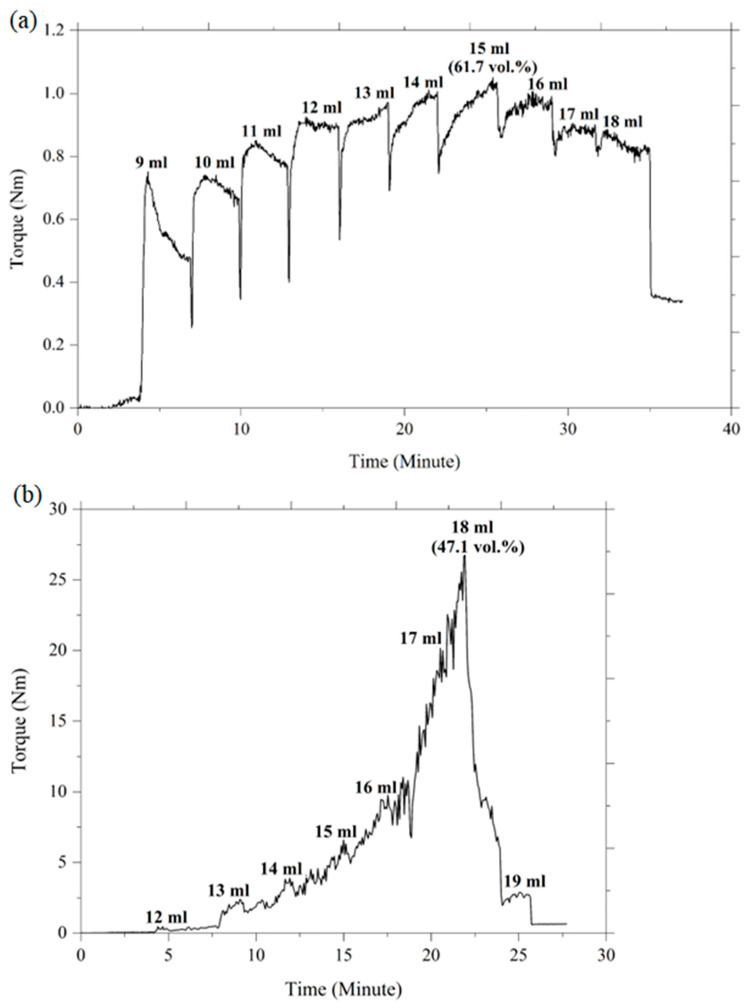
Torque evolution over time of the (**a**) HA and (**b**) 3YSZ powders with increasing oil volumes.

Powder loading is regarded as a crucial feature that has a substantial impact on the success of the subsequent stages of 2C-µPIM. Considering the challenges of high and low powder loadings, as discussed earlier, this present study selected the optimal loading of ceramic powder to produce micro-sized components that were free of defects and deformities. Typically, the optimal powder loading is determined by the CPVC. In the current investigation, oleic acid oil was progressively added to vary the mixing torque so as to determine the CPVC of the HA and 3YSZ powders. The torque was continuously evaluated and recorded using the Brabender mixer in conjunction with the torque rheometer. Figure 5 demonstrates that the addition of the oil to the mixture only led to a slight increase in the torque during the start-up phase. The oil was absorbed by the layer of powder particles, and clusters were formed as the mixing torque stabilized. The augmented clusters promoted a significant increase in torque, which remained unstable at the specific CPVC point while the oil was being gradually introduced to the mixture. According to Figure 5, the torque for the HA and 3YSZ powders increased to a maximum value of 1.01 Nm and 26.8 Nm after the addition of 15 mL and 18 mL of oil, respectively. The further addition of liquid to the mixture resulted in the dilation of the solid structure, an increase in the particle-to-particle distance, and a decrease in the torque [9]. The CPVC percentage at the greatest torque was determined by implementing the following equation:

(1) $$CPVC\;\left(\%\right)=\frac{Volume\;of\;powder}{Volume\;of\;powder+Volume\;of\;oil}\times 100$$

When 15 mL of oleic acid oil was added to the HA powder, the measured CPVC was 61.7 vol%; however, when 18 mL of oil was added, the CPVC of the 3YSZ powder was 41.7%. It was evident that the CPVC value for 3YSZ was significantly lower than that of HA. This could be attributed to the higher surface area of the submicronic 3YSZ powder particles, which necessitated a higher fraction of oil to completely coat each particle [41]. Usually, the optimal powder content is estimated to be a power loading that is 2 to 5 vol% below the CPVC value [23]. Therefore, the selected powder loadings for HA and 3YZ in this experiment were 60 vol% and 45 vol%, respectively.

### 3.2. Preparing the Feedstock

**Figure 6:**
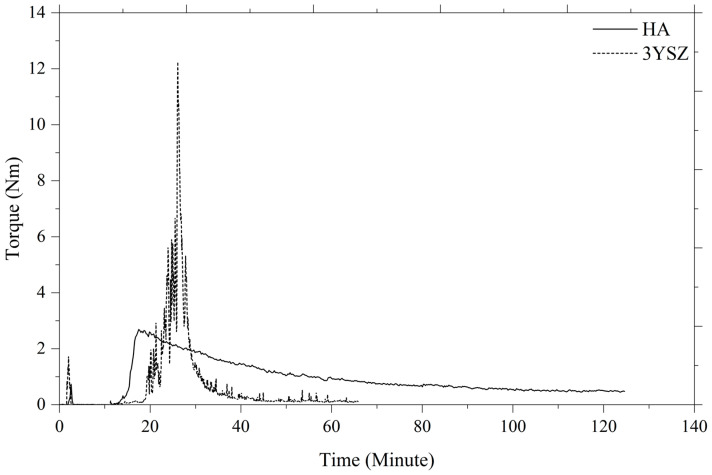
Mixing curves of the HA and 3YSZ feedstocks.

**Figure 7:**
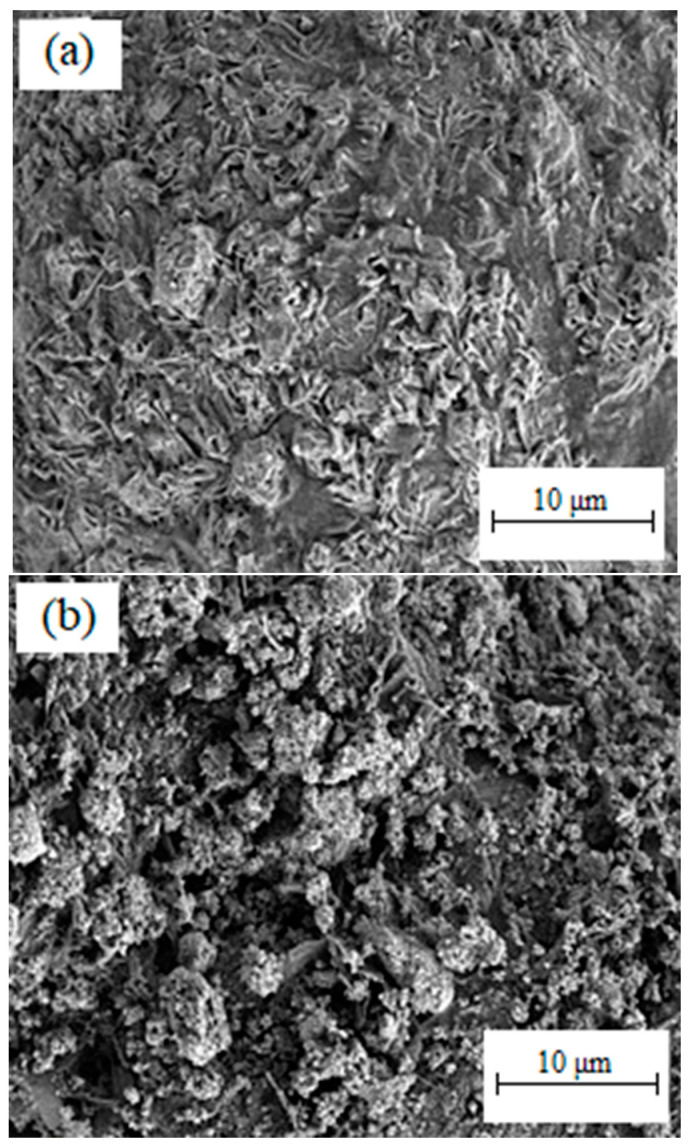
FESEM image of the feedstocks: (**a**) HA and (**b**) 3YSZ.

A critical step in 2C-µPIM, where the powder is mixed with a multi-component binder system, is the fabrication of the feedstock. The quality of the prepared feedstock has a significant impact on the next steps in the process, namely, micro-injection molding, debinding, and sintering. Some of the well-known factors that profoundly affect the quality of the feedstock are the size of the powder particle, powder loading, mixing temperature, mixing time, and shear rate [42]. In this investigation, HA 60 vol% and 3YSZ 45 vol% powder loadings were separately mixed into an LDPE and palm stearin binder system to achieve homogeneous feedstocks. The formation of the HA and 3YSZ feedstocks was performed according to their weight using their constituents’ theoretical densities. In order to obtain the feedstocks in a well-balanced manner, the binder and powder weight fractions were estimated. In this research work, the mixing temperature (150 °C) exceeded the maximum melting temperature (109.2 °C) but did not reach the lowest decomposition temperature (355.8 °C) of the binder system’s components. The proper selection of the mixing temperature not only facilitated melting the binders thoroughly, but also prevented the degradation of the binders. The mixing curves for the HA and 3YSZ feedstocks are displayed in Figure 6. The maximum torque values were 2.69 Nm and 12.24 Nm for the HA and 3YSZ feedstocks, respectively. It is noted in the figure that the 3YSZ feedstock had a higher torque in the start-up phase of mixing compared to the HA feedstock. This could be attributed to the need for an elevated torque to decrease the clusters of agglomerations in the submicronic particles of the 3YSZ powder [14]. The homogeneity of the HA and 3YSZ feedstocks was ensured by the steady torque that was used for mixing at that time, following its preliminary adjustment. The use of an inhomogeneous feedstock frequently leads to a number of challenges, including powder–binder separation and formation of defects in the green part [20,23,42]. Therefore, it is important to employ homogeneous feedstocks during the 2C-µPIM process. The FESEM micrographs of the HA and 3YSZ feedstocks in Figure 7 show that the particles of the HA and 3YSZ powder were completely enclosed by the binders, thus substantiating the homogeneity of the prepared feedstocks.

### 3.3. Rheology

**Figure 8:**
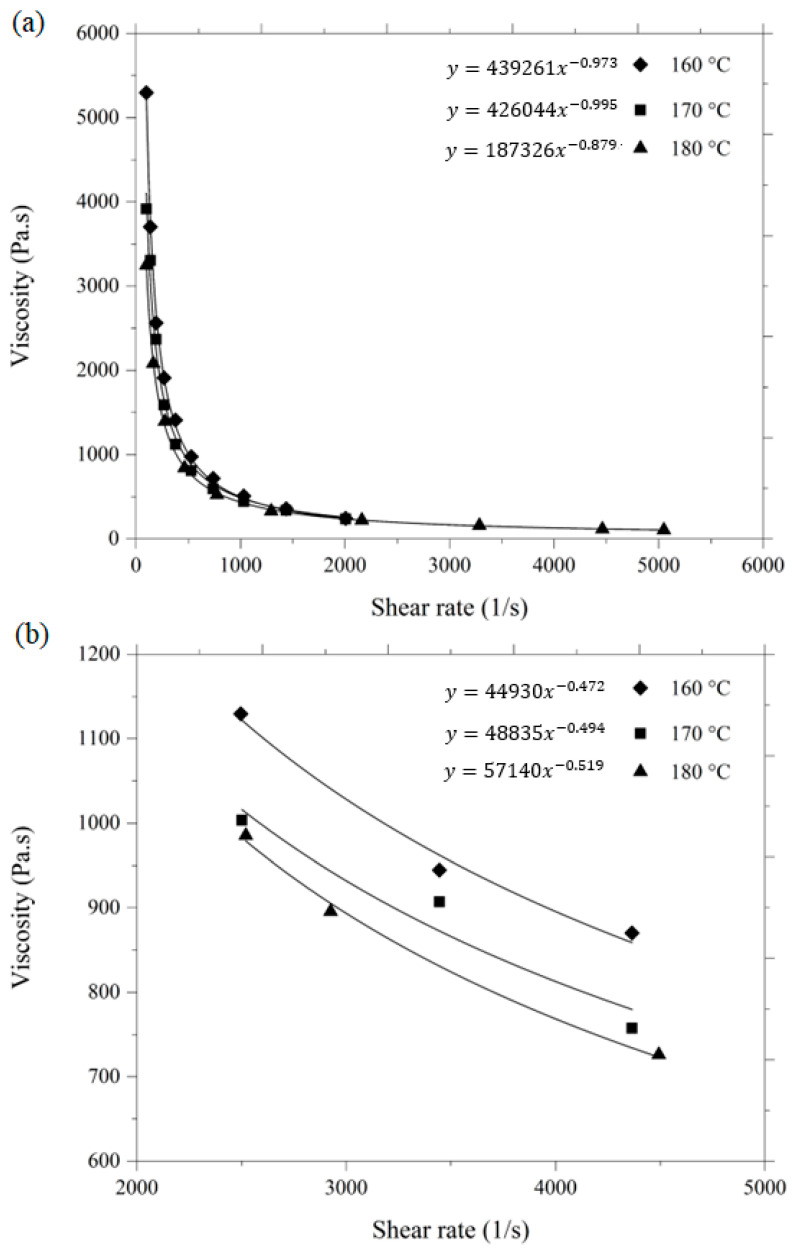
Correlation between the shear rate and viscosity of the (**a**) HA and (**b**) 3YSZ feedstocks.

A rheological study is regarded as a crucial tool for predicting the flow behavior of feedstocks and evaluating how well the mold fills up during the injection molding process [3]. In the current investigation, the rheological characteristics assessment complied with the viscosity profiles of the feedstock in relation to the shear rate and temperature. Figure 8 depicts the graph of the shear rate versus viscosity of the HA and 3YSZ feedstocks at 160, 170, and 180 °C. The viscosity of the HA and 3YSZ feedstocks decreased when the shear rate increased, in a behavior that is known as pseudoplastic or shear thinning. It is a well-known fact that to secure the methodical filling of a small mold cavity during 2C-µPIM and to improve the shape retention capabilities of the injection-molded component, the prepared feedstocks will have to display pseudoplastic behavior [23]. Usually, the dilatant behavior, where the increase in viscosity occurs with the enhancement of the shear rate, promotes segregation of the powders from the binders [9]. According to Figure 8a, the viscosity of the HA feedstock ranged between 507.5–2561.7 Pa·s at 160 °C, and dropped to 157.1–1392.5 Pa·s at 180 °C. At the same time, as seen in Figure 8b, the viscosity of the 3YSZ feedstock ranged between 870.1–1129.3 Pa·s at 160 °C, and reduced to 726.2–985.5 Pa·s at 180 °C. The driving force for such a phenomenon is the loss of attraction within the binder molecules with the enhancement of the temperature [26]. In general, the feedstock can flow much more easily into the mold cavity as the viscosity decreases.

Due to the pseudoplastic behavior of both feedstocks, the following equation can be used to represent the relationship between viscosity $\left(\eta \right)$ and shear rate $\left(\Upsilon \right)$:

(2) $$\eta =K\Upsilon^{n-1}$$

where K and n, respectively, denote a constant and the flow behavior index. Based on Figure 8, n for HA and 3YSZ feedstocks was below a value of 1, revealing that both of the fabricated feedstocks were recognized as exhibiting pseudoplastic behavior. Typically, n represents a measure of the shear sensitivity. The sensitiveness of the viscosity to alterations in shear rate increases with a smaller n value [11]. The range of n for HA and 3YSZ feedstocks was 0.005 < n < 0.121 and 0.0481 < n < 0.528, respectively. In comparison to the 3YSZ feedstock, the HA feedstock was found to have a lower value of n and a higher powder loading, which led to more prominent pseudoplastic behavior. This finding is comparable with one that Sotomayor et al. [24] published in a previous study, where n dropped as powder loading was raised. A feedstock with a lower n value facilitates effective filling of the mold cavity while utilizing less pressure and heat [11].

### 3.4. Micro-Injection Molding

**Figure 9:**
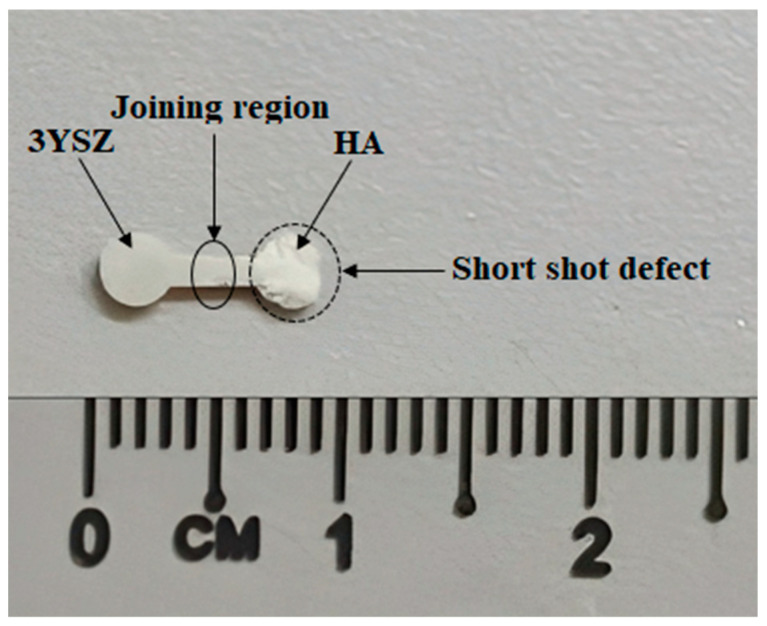
Short-shot defect in bi-material through micro-injection molding.

Micro-injection molding trials were performed on the HA and 3YSZ feedstocks in accordance with the rheological analysis so that appropriate injection molding parameters could be determined. The injection molding parameters that were used to produce the micro-sized bi-material components of HA and 3YSZ are shown in Table 2. As can be seen in the table, the applied melt temperature of 180 °C was much lower than the temperature of 355.8 °C, at which the palm stearin binder began to degrade. The use of mold temperatures below 140 °C frequently revealed short-shot defects in the HA portion of the bi-materials. Figure 9 depicts the short-shot defect that was noticed in a micro-sample at 135 °C. The fundamental cause of this occurrence is the instantaneous cooling of HA feedstock during micro-injection molding owing to the minuscule dimensions of the mold cavity and the resulting diminution of feedstock flowability at mold temperatures below 140 °C [23]. According to Fayyaz et al. [26], eliminating short-shot defects in green micro-parts during micro-injection molding through µPIM can be accomplished by narrowing the gap between mold temperature and melt temperature. Due to this, a mold temperature of 140 °C was employed to achieve defect-free samples. A pressure of 12 bar was used, but any pressure above this increased the likelihood of flash defects in the bi-materials.

**Figure 10:**
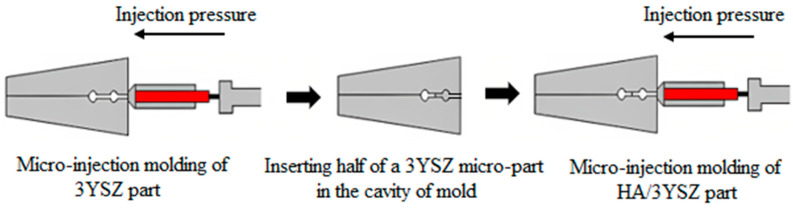
Schematic of processing steps to produce green HA/3YSZ bi-materials.

**Figure 11:**
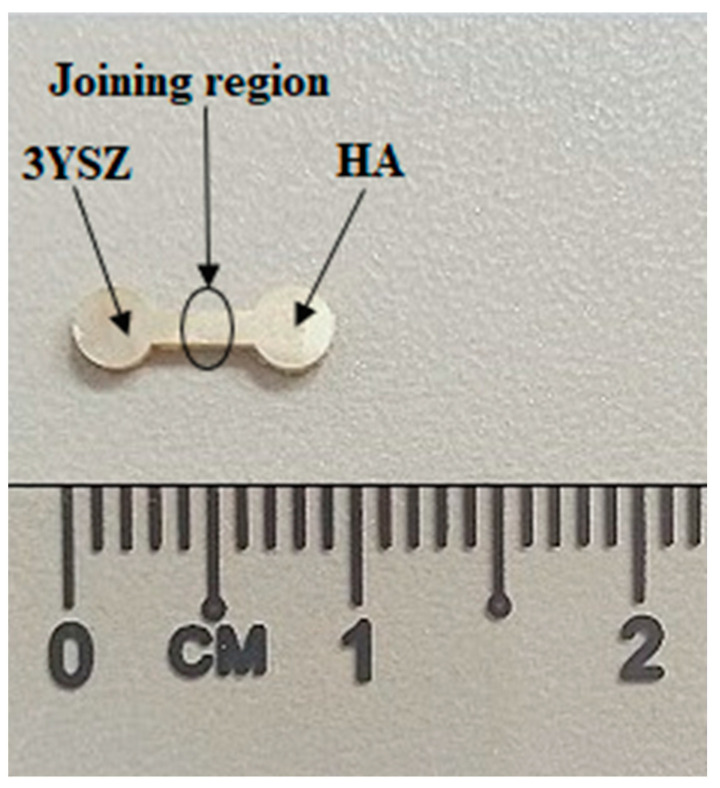
Green micro-sized bi-material component of HA and 3YSZ.

**Figure 12:**
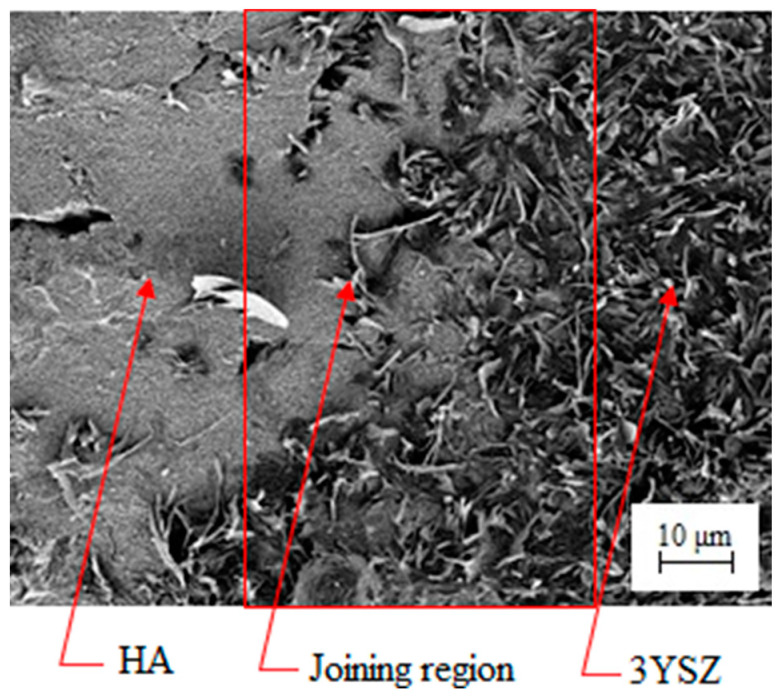
FESEM image of the green HA/3YSZ micro-part.

In this study, green HA/3YSZ micro-components were produced using the sequential technique, as schematically represented in Figure 10. At the beginning of the process, the feedstock of the 3YSZ was inserted into the mold cavity as part of the overall mechanism. After that, the green micro-sized 3YSZ component that was obtained was split into two, and the semi part was again positioned into the mold cavity. Then, the HA feedstock was injected over the 3YSZ feedstock to obtain an interlocked assembly or bi-material. The mold was thoroughly cooled immediately following the injection process and before extracting the part from the mold to ensure that the bi-materials had reached the requisite strength. Figure 11 illustrates the green HA/3YSZ micro-part fabricated using the HA and 3YSZ feedstocks. According to the FESEM image of the component, as depicted in Figure 12, the interface of the interlocked components was made up of HA and 3YSZ. The powder particles in the region joining the green part were suitably covered with binders. Moreover, no defects, cracks, or gaps were observed in that region.

### 3.5. Debinding

**Figure 13:**
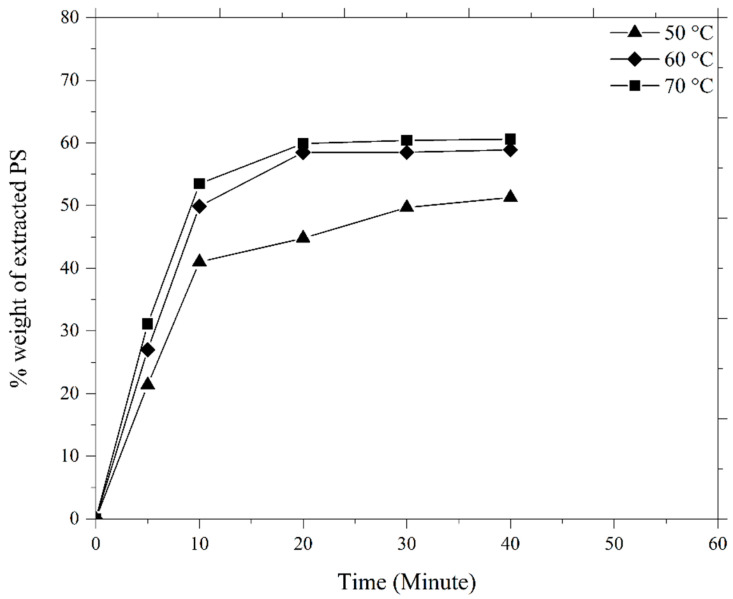
Palm stearin binder loss during the solvent debinding of micro-sized HA/3YSZ components.

**Figure 14:**
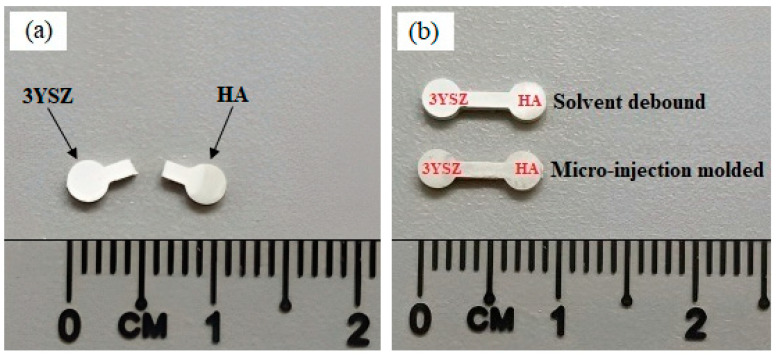
(**a**) Failure of the joining in bi-material during solvent debinding at 80 °C, and (**b**) comparison between micro-injection molded and solvent debound samples in terms of physical changes.

**Figure 15:**
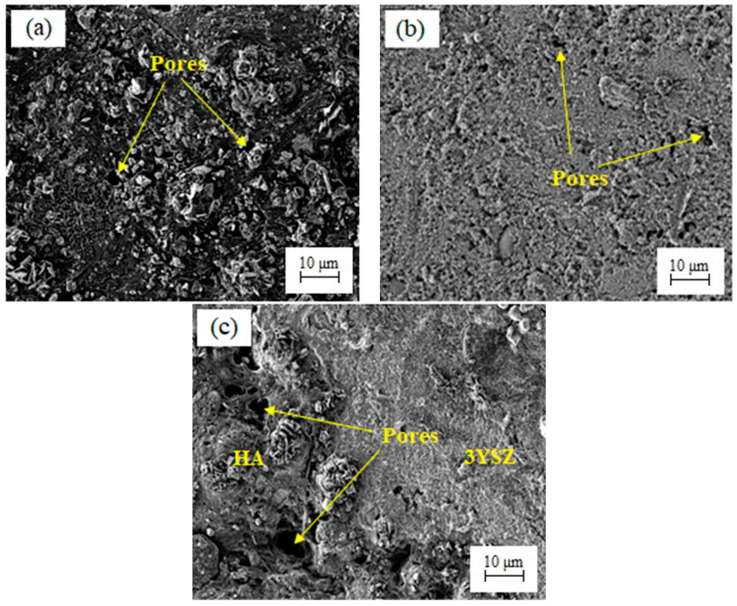
FESEM micrographs of the solvent-debound HA/3YSZ micro-sized component: (**a**) HA portion, (**b**) 3YSZ portion, and (**c**) joining region.

The debinding process that was performed on the green HA/3YSZ micro-parts to remove the LDPE and palm stearin binders involved two steps; namely, thermal debinding and solvent debinding. During solvent debinding, when the green parts were placed in the solvent immersion, the palm stearin binder began to integrate with the solvent to form a swelling gel. Once an adequately high solvent concentration was reached, the gel started to dissolve [43,44]. As the binder of palm stearin had been partially removed, the micro-sized samples contained pores. As the debinding temperature and time increased, these pores grew to encompass the interior of the sample [14,43]. Figure 13 depicts the palm stearin binder volume that was extracted over time from the HA/3YSZ micro-sized specimens at 50, 60, and 70 °C. As seen, as the time and solvent debinding temperature increased, the palm stearin’s mass loss also increased. Moreover, the rate of elimination of the soluble binder was quite fast in the first 20 min and became sluggish or sporadically constant over the next 20 min. As seen in Figure 13, as the debinding temperature of the solvent increased from 50 to 70 °C, the rate at which the palm stearin was eliminated increased from 51.3 to 60.6%. Typically, the diffusion mechanism serves as the sole basis for the regulation of the solvent debinding process. The temperature impacts the rate of diffusion, and an increase in temperature will result in a greater elimination of the binder due to the higher diffusion rate [45]. A solvent debinding temperature of 70 °C was selected because at 80 °C, joining failure between the HA and 3YSZ occurred in some bi-materials, as shown in Figure 14a, which could be attributed to polymer softening and quick removal of palm stearin. The micro-injection molded HA/3YSZ component and the component processed through solvent extraction at 70 °C had similar dimensions, as can be seen in Figure 14b. The FESEM images of the bi-material after solvent debinding are shown in Figure 15, where it can be seen that a reasonable amount of palm stearin was eliminated from the joining area and other portions of the sample, leaving an adequate open-pore structure. Such an open-pore structure was necessary to completely remove the LDPE, which is insoluble, during thermal debinding.

**Figure 16:**
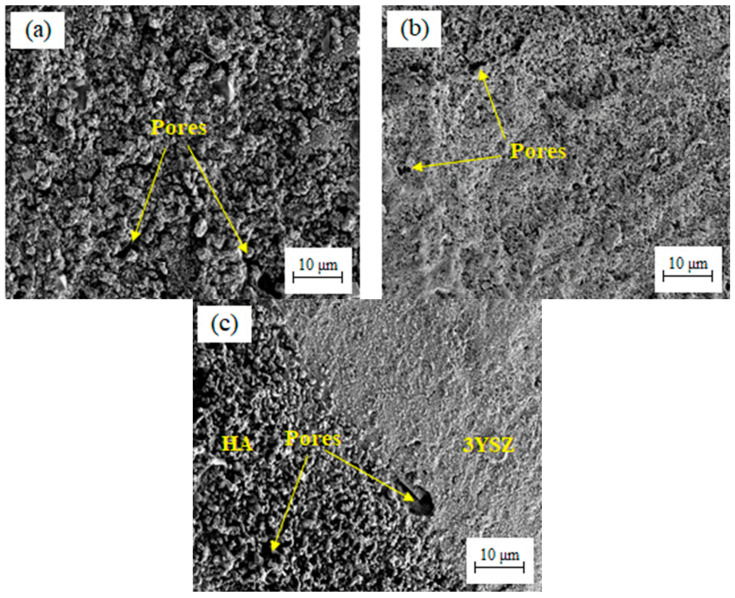
FESEM micrographs of the thermally debound HA/3YSZ micro-sized component: (**a**) HA portion, (**b**) 3YSZ portion, and (**c**) joining region.

Thermal debinding is widely used as the primary method for getting rid of organics before sintering [46]. In this experiment, the thermal debinding method was employed to eliminate the remaining palm stearin and LDPE binders. Here, the open pores, which were visible during the debinding of the solvent, facilitated the removal of the gaseous decomposed products [14]. Thermally debinding the samples eliminated 93 to 95% of the binder system while the remnants were removed via sintering. In order to prevent the development of cracks in debound HA/3YSZ micro-samples, very slow heating rates (0.1 and 0.25 °C/min) were implemented progressively [20,23]. The bi-materials become incredibly fragile after the elimination of the binder system; therefore, they should be managed cautiously. Figure 16 depicts the FESEM images of the HA/3YSZ micro-component post-thermal debinding. As seen, the binders were almost entirely removed from the HA and 3YSZ portions as well as the joining region of the bi-material.

### 3.6. Sintered HA/3YSZ Micro-Part

**Figure 17:**
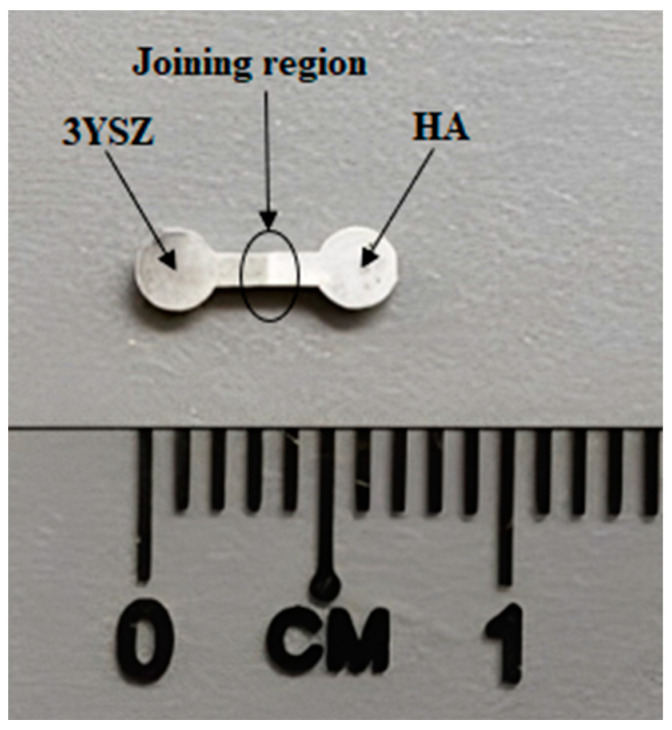
Image of the sintered HA/3YSZ micro-sized component.

The image of an HA/3YSZ micro-sized component sintered at 1300 °C is shown in Figure 17. During the sintering process, densification and linear shrinkage showed up as the ceramic powder particles fused together and filled the vacant space left by the elimination of the binder [23,47,48]. The maximum density of the sintered bi-material, 3.91 g/cm^3^, was 96.3% of the theoretical density. Heaney [20] reported that PIM-based components often need to have a relative density of at least 95%. This viewpoint enables us to accept the highest relative density of the sintered HA/3YSZ micro-sample processed through 2C-µPIM found in this study as reasonable. There was a linear shrinkage ranging from 13 to 17% when the sintered part was compared to the green part. The typical linear shrinkage range of the sintered micro-samples, according to Piotter [49], is 15 to 22%. In this experiment, the feedstocks produced utilizing the optimal powder loading led to sintered bi-materials with a decent shrinkage range. Another study will be conducted in the future to examine the bonding and mechanical properties of micro-sized HA/3YSZ components that have been sintered.

## 4. Conclusions

This research work aimed to use the 2C-µPIM process to produce green HA/3YSZ micro-sized components that were free of defects and to perform solvent and thermal debinding on them to obtain debound parts for a sintering operation. The following conclusions were drawn in light of the experimental findings and the previously mentioned discussions:

- The optimal powder loadings were determined based on an analysis of the CPVC. Homogeneous HA and 3YSZ feedstocks with 60 and 45 vol% powder loadings, respectively, were successfully fabricated. Both of the feedstocks exhibited pseudoplastic behavior, ensuring efficient filling of the small mold cavity during the injection process.
- A sequential mechanism was used to fabricate the HA/3YSZ micro-parts. Mold temperature is a key factor in the injection molding of HA/3YSZ micro-components. Short-shot defects have been found to frequently occur in bi-materials when a mold temperature of less than 140 °C is used. Hence, this present study used a mold temperature of 140 °C throughout the entire micro-injection molding process to obtain defect-free green samples.
- The temperature for solvent debinding was set at 70 °C, the temperature at which the largest quantity of palm stearin was removed. Thermal removal was found to eliminate approximately 93 to 95% of the binder system, leaving only 5 to 7% in the HA/3YSZ micro-parts pre-sintering. Lastly, the sintered HA/3YSZ micro-parts exhibited the highest density of 3.91 g/cm^3^ and shrank linearly between 13 and 17% at a sintering temperature of 1300 °C.

## Figures and Tables

**Table 1:** Characteristics of the binders.

Binders	Chemical Designation	Melting Point (°C)	Decomposition Temperature Range (°C)
LDPE	$(C_{2}H_{4})n$	109.2	397.8–501.4
Palm stearin	CH_3_(CH_2_)_14_COOH	49.8	355.8–465.9

**Table 2:** Injection parameters used to fabricate the micro-sized green HA/3YSZ components.

Melt Temperature (°C)	Mold Temperature (°C)	Injection Pressure (bar)	Holding Pressure (bar)	Injection Time (s)
180	140	12	12	7

## Data Availability

Data that supporting the results of this study are available from the first author.
